# Nuclear Envelope-Associated Chromosome Dynamics during Meiotic Prophase I

**DOI:** 10.3389/fcell.2017.00121

**Published:** 2018-01-09

**Authors:** Xinhua Zeng, Keqi Li, Rong Yuan, Hongfei Gao, Junling Luo, Fang Liu, Yuhua Wu, Gang Wu, Xiaohong Yan

**Affiliations:** Oil Crops Research Institute of the Chinese Academy of Agricultural Sciences, Key Laboratory of Biology and Genetic Improvement of Oil Crops, Ministry of Agriculture, Wuhan, China

**Keywords:** nuclear envelope, chromosome dynamics, meiosis prophase I, SUN proteins, KASH proteins, meiotic modification, cytoplasmic adaptors, nucleoplasmic adaptors

## Abstract

Chromosome dynamics during meiotic prophase I are associated with a series of major events such as chromosomal reorganization and condensation, pairing/synapsis and recombination of the homologs, and chromosome movements at the nuclear envelope (NE). The NE is the barrier separating the nucleus from the cytoplasm and thus plays a central role in NE-associated chromosomal movements during meiosis. Previous studies have shown in various species that NE-linked chromosome dynamics are actually driven by the cytoskeleton. The linker of nucleoskeleton and cytoskeleton (LINC) complexes are important constituents of the NE that facilitate in the transfer of cytoskeletal forces across the NE to individual chromosomes. The LINCs consist of the inner and outer NE proteins Sad1/UNC-84 (SUN), and Klarsicht/Anc-1/Syne (KASH) domain proteins. Meiosis-specific adaptations of the LINC components and unique modifications of the NE are required during chromosomal movements. Nonetheless, the actual role of the NE in chromosomic dynamic movements in plants remains elusive. This review summarizes the findings of recent studies on meiosis-specific constituents and modifications of the NE and corresponding nucleoplasmic/cytoplasmic adaptors being involved in NE-associated movement of meiotic chromosomes, as well as describes the potential molecular network of transferring cytoplasm-derived forces into meiotic chromosomes in model organisms. It helps to gain a better understanding of the NE-associated meiotic chromosomal movements in plants.

## Introduction

Meiosis has the following characteristics, one round of DNA replication and two rounds of chromosome separation (Roeder, 1997). Prophase I is the longest and most complex phase of meiosis, which is vital to ensure the faithful completion of meiosis. A series of chromosome dynamics-associated events such as chromosomal reorganization and condensation, establishment of meiotic-specific chromosome structure, homologous chromosome pairing, and dynamic chromosome movements is closely integrated and finely spatiotemporally controlled during meiotic prophase I (Padmore et al., 1991; Dawe et al., 1994; Hunter and Kleckner, 2001; Blat et al., 2002; Borner, 2006; Golubovskaya et al., 2006; Kleckner, 2006; Zickler, 2006; Tiang et al., 2012). During meiosis, telomeres attach to the nuclear envelope (NE), which in turn drives chromosome movement (Tiang et al., 2012). The NE is a highly conserved eukaryotic structure that protects DNA from enzymatic degradation (Stewart et al., 2007; Wilson and Dawson, 2011). Recent studies have shown that the NE fulfills distinct functions by regulating sets of the proteins that are embedded in the NE. Furthermore, the NE is a crucial determinant for reproduction and fertility; its particular components, the Klarsicht/ANC-1/Syne-1 homology (KASH) proteins and Sad-1/UNC-84 homology (SUN) proteins, play a key role in meiotic chromosome movements (Razafsky and Hodzic, 2009; Kracklauer et al., 2013; Subramanian and Hochwagen, 2014). Nonetheless, the precise role of the NE in chromosome dynamics remains elusive. Here, we review recent studies on meiosis-specific constituents and modifications involving the NE and related nucleoplasmic/cytoplasmic adaptors, as well as propose a molecular network of cytoplasm-derived forces that influence NE-linked meiotic chromosomal movements.

## An overview of the NE structure

**Figure 1:**
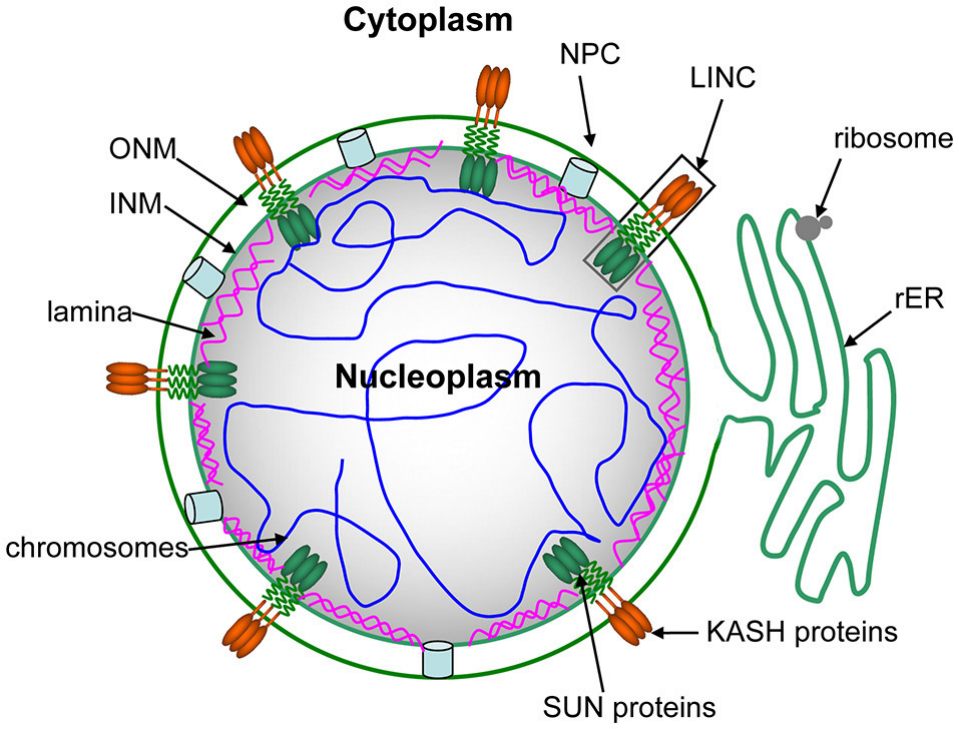
The interphase structure of the NE. The NE consists of the inner nuclear membrane (INM), outer nuclear membrane (ONM) and the perinuclear space (PNS). The NE is embedded with nuclear pore complexes (NPCs), SUN proteins in the INM and KASH proteins in the ONM. LINC complexes are made of SUN proteins and KASH proteins, transferring cytoplasm-derived forces inti the chromosomes in the nucleoplasm. The ONM facing the cytoplasm is connected with the rough endoplasmic reticulum (rER). The nuclear lamina is a protein network that is situated close to the INM nucleoplasmic side. In plants, little is known about the nuclear lamina. However, electron microscopy has revealed there is an INM-associated dense meshwork, similar to the animal lamina.

In eukaryotes, the nucleus is a characteristic feature of eukaryotic cells that is enclosed by the NE. Figure 1 shows the structure of the NE during interphase. The NE is a highly conserved eukaryotic double membrane that separates and protects the genetic material of cells (Stewart et al., 2007; Wilson and Dawson, 2011). The general structure of the NE consists of the inner nuclear membrane (INM), outer nuclear membrane (ONM), and the perinuclear space (PNS), which is about 50 nm in thickness and situated between the INM and ONM (Figure 1). The double nuclear membranes are connected by nuclear pore complexes (NPCs) and linkers of nucleoskeleton and cytoskeleton (LINC) complexes (Figure 1; Crisp et al., 2006). NPCs serve as the fusion site of the INM and ONM and form transport channels for macromolecules that move to and from the nucleus and cytoplasm. LINCs stabilize the structure of the NE, play important roles in cell division, and establish cellular polarity, fertilization, cellular migration, and differentiation by connecting the INM and ONM (Crisp et al., 2006; Rothballer et al., 2013; Sosa et al., 2013). However, despite these junctions, the ONM and INM are still divergent. The ONM is a specialized extension of the endoplasmic reticulum (ER), which is studded with ribosomes that facilitate protein synthesis (Park and Craig, 2010). The ONM also binds cytoskeletal components such as microtubules (MTs), as well as acts as a nucleation center of MTs during cell division (Han and Dawe, 2011; Masoud et al., 2013). A series of proteins in the INM interact with various nuclear constituents, including chromosomes and the nucleoskeleton, to ensure the link between the NE and the corresponding nuclear materials (Starr, 2009; Bickmore and van Steensel, 2013). The nuclear lamina as a protein network juxtaposed to the INM nucleoplasmic side. However, currently understanding of the nuclear lamina in plants is limited. An INM-linked dense meshwork was founded in plants by electron microscopy, that is similar to animal laminae (Ciska and de la Espina, 2014).

Recent studies have shown that the NE is not only a physical nucleocytoplasmic barrier, but also a multifunctional platform (Fransz and de Jong, 2011; Gross and Bhattacharya, 2011). The NE thus allows specific proteins to be embedded in the ONM and INM, respectively, thereby establishing specific cytoplasm-facing and nucleoplasm-facing functions. A collection of specific integral membrane proteins in the NE include nuclear pore complexes (NPCs), SUN proteins (Razafsky and Hodzic, 2009; Starr and Fridolfsson, 2010) in the INM, and KASH proteins (Wilhelmsen et al., 2006; Rothballer and Kutay, 2013) in the ONM. SUN proteins and KASH proteins form LINC complexes (Crisp et al., 2006). Thus, animal NE proteins transport nucleocytoplasmic macromolecules, are involved in chromosomal dynamics, regulate transcription, and induce aging and nuclear migration (Gruenbaum et al., 2005; Andres and Gonzalez, 2009; Hetzer and Wente, 2009; Starr, 2009). Furthermore, certain NE components play a key role in chromosome pairing and synapsis of homologs during meiosis (Subramanian and Hochwagen, 2014). The LINC complex is an important NE component that has been implicated in the directed movement of meiotic chromosomes within the nucleus (Razafsky and Hodzic, 2009; Kracklauer et al., 2013).

## Chromosome dynamics in meiosis

DNA is replicated once, but chromosomes are segregated twice during meiosis (Roeder, 1997). Meiotic divisions are subdivided into meiosis I and meiosis II. Homologous chromosomes are separated in meiosis I, and sister chromatids are segregated from each other in meiosis II. A series of coordinated processes are required during the two meiotic divisions. Prophase I, metaphase I, anaphase I, and telophase I occur in meiosis I. Prophase I as the longest and most complex phase and is further subdivided into five distinguished stages according to the degree of chromatin condensation. The stages in succession are leptotene, zygotene, pachytene, diplotene, and diakinesis (Baarends and Grootegoed, 2003; Wijnker and Schnittger, 2013).

Chromosome dynamics including reorganization and condensation of chromosomes, homologous chromosome pairing, chromosome movements, and establishment of meiosis-specific chromosome structure occur during prophase I of meiosis (Tiang et al., 2012). Homologous chromosome pairing (Dawe et al., 1994) is tightly associated with the process of meiotic recombination (Tiang et al., 2012). Meiosis involves unique chromosome dynamic processes such as pairing/ synapsis and recombination of homologs that occur during meiotic prophase I, as have been extensively characterized in model systems involving *Saccharomyces cerevisiae, Schizosaccharomyces pombe*, and *C. elegans* (Hiraoka and Dernburg, 2009; Koszul and Kleckner, 2009). These meiosis-specific events are closely integrated and finely controlled temporally and spatially (Padmore et al., 1991; Hunter and Kleckner, 2001; Blat et al., 2002; Borner, 2006; Kleckner, 2006; Zickler, 2006). Synapsis and recombination ensure the establishment of chiasmata that hold homologous chromosomes together, thereby facilitating correct segregation (Tiang et al., 2012).

## Telomere movements at the NE during meiosis

**Figure 2:**
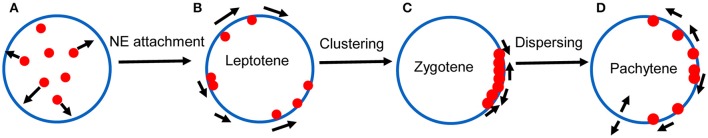
Telomere movement at the NE during meiotic prophase. The four different movement classes are indicated as **(A–D)**. Red dots indicate the positions of the telomeres relative to the NE. The relative direction of telomeric movements is indicated by black arrows. **(A)** Telomeres scattering in the nucleus move to the NE at the onset of leptotene stage. **(B)** Telomeres are tethered to the NE and stably connected to it at the late leptotene stage. The telomere clustering starts in the late leptotene stage, always overlaps with the zygotene stage and usually persists until pachytene. **(C)** The tightest clustering of telomeres is usually observed at the zygotene stage. **(D)** At pachytene, telomeres are motile and scattered over the NE again, while additional nuclear deformations and rotations occur (black arrows). For further information please see the Scherthan (2007).

Telomeres are blocks of highly conserved repetitive DNA sequences at chromosome ends that protect chromosomes from nucleolytic degradation and fusion. The behavior of centromeres and telomeres largely controlls chromosomal dynamics of prophase I (Siderakis and Tarsounas, 2007). Previous studies have shown in various species that the cytoskeleton induces chromosomal movements using telomere-NE attachments (Bhalla and Dernburg, 2008; Koszul and Kleckner, 2009; Sheehan and Pawlowski, 2009; Woglar and Jantsch, 2014). During meiosis prophase I, telomere positions undergo dynamic changes, including telomeric attachment, clustering, dispersal, and redistribution across the nuclear periphery (Figure 2). During meiotic interphase, telomeres are distributed across the nucleolus instead of the NE. Prior to pairing, telomeres attach to the NE at the onset of leptotene stage. As leptotene proceeds, telomeres are attached to the NE and are stably linked to it. These tethered telomeres move within the INM and gather at a certain region, creating a characteristic flower-like structure, known as the bouquet of telomeres (Bass et al., 2000; Golubovskaya et al., 2002; Harper et al., 2004; Richards et al., 2012). Telomere clustering starts at the late leptotene stage, always overlaps with the zygotene stage, and usually persists until pachytene (Bass, 2003). The telomere bouquet always appears during the zygotene stage, after which telomeres are then scattered again. Despite telomere clustering may be observed at the early pachytene stage, if homologous chromosomes are completely paired at the end of pachytene, telomeres are dispersed evenly across the NE again while additional nuclear deformations and rotations occur.

The characteristic telomere-guided chromosome movements are an evolutionarily highly conserved hallmark of meiotic prophase I (Scherthan et al., 1996; Koszul and Kleckner, 2009). The telomere “bouquet” stage has been observed in all organisms studied regardless of whether they have big (maize) or small (fission yeast) genomes (Scherthan, 2001), except *C. elegans* and *Drosophila*, which both employ non-canonical methods of homology searching (Mckee, 2004).

## Functional significance of the telomere bouquet

Bouquet formation of telomeres feature chromosomal movements within the NE, which might facilitate homologous chromosome pairing and synapsis (Scherthan, 2001; Lee et al., 2012). Several lines of evidence show that one of the most likely functions of the bouquet is to warrant the efficient initiation of pairing and synapsis of between homologous chromosomes (Tabata, 1962; Carlton and Cande, 2002; Moens et al., 2011). Mutants with defects in bouquet generation always show defects in chromosome pairing, which suggests the possible role of the bouquet in chromosome pairing (Harper et al., 2004; Klutstein and Cooper, 2014). Several mutants, for example, *plural abnormalities of meiosis 1* (*pam I*) (Golubovskaya et al., 2002), *desynaptic 1* (*dsy1*) (Bass et al., 2003), and *poor homologous synapsis1* (*phs1*) (Pawlowski et al., 2004) exhibit significant defects in homologous pairing in maize. Correspondingly, clusters of telomeres persist in pairing-defective *spo1*1 mutants of *Sordaria* and *S. cerevisiae* (Trelles-Sticken et al., 1999). Therefore, it seems likely that the bouquet physically brings homologous chromosomes into close proximity at a certain region of the NE, supporting homologous chromosome pairing and synapsis, double-strand break (DSB) repair, and recombination (Scherthan et al., 1996; Bass et al., 2000), thereby preventing and dissolving heterologous associations of non-homologous chromosomes (Zickler and Kleckner, 1998; Moens et al., 2011). However, the actual function of the meiotic bouquet is still not entirely clear.

## LINC complexes

It has been shown in several species that the cytoskeleton induces dynamic motility of chromosomes via telomere-NE attachments (Bhalla and Dernburg, 2008; Koszul and Kleckner, 2009; Sheehan and Pawlowski, 2009; Woglar and Jantsch, 2014). The NE is the barrier separating the nucleus from the cytoplasm that plays a central role in the NE-associated chromosomal movements. Significantly, NE-linked chromosome dynamics are actually driven by the cytoskeleton during meiotic progression (Trelles-Sticken et al., 1999; Conrad et al., 2008; Koszul et al., 2008; Lee et al., 2012). The implication here is that there must be mechanisms that transmit cytoskeletal forces across the NE to individual chromosomes. The special double-layer-membrane structure of the NE raises the question of how can various regions of chromosomes, telomeres in particular, be physically connected to the cytoskeleton during meiosis. Because the NE remains intact during the process of synapsis, there has to be a molecular machinery spanning both the INM and ONM and interacting with chromatin and other cytoskeletal components, respectively. The LINC complexes consist of SUN domain family proteins in the INM and KASH domain homology proteins in the ONM (Burke and Roux, 2009; Razafsky and Hodzic, 2009; Starr and Fridolfsson, 2010).

The LINC complexes span the INM and ONM and form the bridge between the nucleoskeleton and the cytoskeleton through the SUN-KASH domain interaction in the NE lumen (Razafsky and Hodzic, 2009; Starr and Fridolfsson, 2010; Kim et al., 2015). In this way, mechanical forces from the cytoskeleton are directly transduced to the NE and then into chromosomes. A chain of interactions from the cytoskeletal elements to the nucleoskeleton as follows, various components of the cytoskeleton interact with the cytoplasmic domains of KASH proteins, which in turn induces SUN proteins in the INM to interact with KASH proteins at their C-termini in the PNS and with specific nuclear contents at the N-termini in the nucleoplasm (Haque et al., 2006; Bone et al., 2014). The LINC complexes are responsible for the transfer of this force across the nuclear envelope and enable a direct communication and connection between nuclear and cytoplasmic content.

## SUN domain proteins

### Molecular characteristics of sun proteins

SUN proteins as important INM-integral components of LINC complexes that exhibit highly conserved structure and function (Starr, 2009). SUN proteins comprise an N-terminal region and a C-terminal region that are separated by one or more transmembrane domains (TMDs) (Tzur et al., 2006; Worman and Gundersen, 2006). The N-termini of SUN proteins are variable and directly or indirectly interact with lamins, which are the components of the nucleoskeleton (Lee et al., 2002; Crisp et al., 2006; Haque et al., 2006; Bone et al., 2014) and tether chromosomes to the nuclear periphery (Bupp et al., 2007; King et al., 2008; Morimoto et al., 2012; Link et al., 2014). The C-terminal region contains the well-conserved SUN domain, which extends into the PNS that interacts with KASH proteins. Most SUN proteins have coiled-coil domains (CCDs) at their N-termini, which facilitate in domain trimerization (Sosa et al., 2012; Zhou et al., 2012b).

Two divergent classes of SUN proteins have been identified by homology searching in plants: classical SUN proteins which contain SUN domains at the C-terminus (Murphy et al., 2010), and a second group of SUN proteins, with SUN domains in the center of the SUN protein, and thus designated as mid-SUN proteins (Murphy et al., 2010). The function of mid-SUN proteins is far less well-understood than the Cter-SUNs. Mid-SUN proteins differ from Cter-SUN proteins in both structure and localization. Mid-SUN proteins frequently contain three TMDs and plant mid-SUN proteins usually contain a conserved PM3-associated domain (PAD) (Murphy et al., 2010; Graumann et al., 2014). In addition, mid-SUN proteins are located in both the NE and the ER (Murphy et al., 2010; Graumann et al., 2014).

### Members and functions of sun proteins

SUN domain proteins have been identified in various species (Table 1). Three *Arabidopsis* SUN proteins (AtSUN3, AtSUN4, and AtSUN5) and three maize SUN proteins (ZmSUN3, ZmSUN4, and ZmSUN5) belong to the mid-SUN group (Murphy et al., 2010; Murphy and Bass, 2012; Graumann et al., 2014). The presence of several SUN protein members in a single organism (often at least five in humans) and their ability to form multimers implicate these are involved in a wide range of important cellular functions. Reports have shown that SUN proteins are implicated in interactions with lamins, nuclear positioning, spindle architecture, apoptosis, centrosome linkage to the nucleus, and maintenance of even spacing between the INM and ONM (Table 1). In addition, SUN proteins are required in a number of systems to attach telomeres or pairing centers to the NE during meiosis (Chikashige et al., 2006; Ding et al., 2007; Penkner et al., 2007; Conrad et al., 2008; Koszul et al., 2008). For example, SUN1, SUN2, Sad1, and Mps3 tether chromosomes to the nuclear periphery by interacting with telomere-binding proteins (Bupp et al., 2007; King et al., 2008; Morimoto et al., 2012; Link et al., 2014). The SUN protein trimer can usually bind three KASH domains of KASH homology proteins in the PNS (Sosa et al., 2012; Zhou et al., 2012a). In maize, ZmSUN2 produces a unique belt-like structure at the NE that undergoes remarkable dynamic changes during meiosis (Murphy et al., 2014). Accordingly, AtSUN1 and AtSUN2 have been localized to meiotic prophase I-specific regions (Varas et al., 2015). In maize, ZmSUN3 as a mid-SUN protein, has been supposed to play an important role in meiotic divisions (Murphy and Bass, 2012). Of the five identified SUN proteins of mammals, SUN1 and SUN2 proteins have been demonstrated to be the only ones that are also expressed in meiotic cells, thereby indicating dual somatic and meiotic functions (Schmitt et al., 2007; Chi et al., 2009; Yu et al., 2011). To date, studies involving SUN1- and SUN1/SUN2-deficient mice have revealed that although SUN2 functions in part similarly to SUN1 in meiosis, SUN2 can not effectively compensate for the loss of SUN1 in meiosis (Schmitt et al., 2007; Chi et al., 2009; Lei et al., 2009). However, a single mutation for either *SUN1* or *SUN2* genes has no effect on reproduction or meiosis in *A. thaliana* (Varas et al., 2015). Several groups have then hypothesized that SUN1 and SUN2 assemble heteromultimeric complexes (Wang et al., 2006; Lu et al., 2008). Taking into account that in mice, SUN2 protein shares its localization with SUN1 protein and meiotic KASH5 protein, it is then speculated that during normal meiosis SUN1 and SUN2 form heterotrimers which interact with KASH5 protein to assemble meiotic LINCs. In the absence of SUN1, LINCs may only consist of SUN2 and KASH5, still attaching telomeres of chromosomes to the NE, yet in a less effective way than complete SUN1/SUN2-KASH5 complexes. And then this could explain the partial redundancy between SUN1 and SUN2 in mice. Further research is required to determine how these SUN family members coordinate in the near future.

**Table 1:** Members and functions of the SUN protein family.

**Members**	**Functions**	**References**
**Mammals**		
SUN1 SUN2	Movement and attachment of telomere in meiosis; nuclear anchorage and migration; integrity of the NE; recruit KASH proteins	Hodzic et al., 2004; Padmakumar et al., 2004; Crisp et al., 2006; Haque et al., 2006; Ding et al., 2007; Zhang et al., 2009; Morimoto et al., 2012
SUN3	Links the nucleus to posterior manchette during sperm head formation	Göb et al., 2010
SPAG4	Not at the NE, function unknown	Shao et al., 1999
SPAG4L	Not at the NE; Links the acrosomic vesicle to the spermatid nucleus; involved in acrosome biogenesis	Frohnert et al., 2011
***Drosophila***		
Klaroid	Nuclear anchorage during Drosophila oogenesis.; nuclear migration	Patterson et al., 2004; Yu et al., 2006; Kracklauer et al., 2007
SPAG4/Giacomo	Not at the NE; involved in centriolar-nuclear attachment during spermatogenesis	Malone et al., 2003
***C. elegans***		
UNC-84	Nuclear positioning; nuclear anchorage and migration	Starr et al., 2001; Starr and Han, 2002
SUN-1/matefin	Links the centrosome to nucleus; homologous chromosome pairing and synapsis in meiosis; apoptosis	Malone et al., 2003; Tzur et al., 2006; Penkner et al., 2009; Sato et al., 2009
***S. pombe***		
Sad1	Spindle architecture; meiotic chromosome pairing and synapsis	Shimanuki et al., 1997; Miki et al., 2004; Chikashige et al., 2006; Ding et al., 2007
***S. cerevisiae***		
Mps3	Linkage to the NE of SPB; SPB duplication; telomere attachment to and clustering within the NE	Jaspersen et al., 2006; Conrad et al., 2008; Wanat et al., 2008; Horigome et al., 2011
***Arabidopsis***		
AtSUN1 AtSUN2	Recruit KASH proteins to the NE; nuclear elongation and movement; meiotic recombination and synapsis	Graumann et al., 2010; Oda and Fukuda, 2011; Zhou et al., 2012a, 2015a,b; Tamura et al., 2013; Varas et al., 2015
AtSUN3, AtSUN4, AtSUN5	Mid-SUN proteins; seed development and involved in nuclear morphology	Graumann, 2014; Zhou et al., 2015b
**Maize**		
ZmSUN1 ZmSUN2	Involved in meiotic telomere dynamics	Murphy et al., 2014
ZmSUN3 ZmSUN4 ZmSUN5	Mid-SUN proteins; ZmSUN3 plays a role in meiosis; ZmSUN4/ZmSUN5: unknown functions	Murphy et al., 2010; Murphy and Bass, 2012
***Dictyostelium***		
Sun-1	Centrosome attachment; genome stability	Xiong et al., 2008

## KASH domain proteins

### Molecular characteristics of KASH proteins

Four criteria were employed to define KASH proteins (Starr, 2011). First, KASH proteins are positioned at the ONM. Second, the C-terminal KASH domain is essential for interaction between KASH and SUN proteins. Third, the KASH domains ensure their localization to the ONM (Crisp et al., 2006). Fourth, N-terminal domains of KASH proteins are not highly conserved and are linked to the cytoskeleton. The KASH domain usually includes a hydrophobic transmembrane domain and a sequence of 6–30 amino acids in the PNS. The perinuclear 6- to 30-amino acid domain of KASH proteins is usually highly conserved, for example, 13 of 20 residues are identical between *C. elegan*s ANC-1 and human Syne/Nesprin-1/-2. The terminal region of the perinuclear sequence of the KASH domain consists of a highly conserved four-amino acid motif PPPX in most animals; however, specifically, the penultimate proline appears to be widely conserved across kingdoms, which is essential in mediating SUN-KASH interaction (Lenne et al., 2000; Razafsky and Hodzic, 2009; Starr and Fridolfsson, 2010; Sosa et al., 2012). Apart from the PPPX motif, the last C-terminal four amino acids of plant KASH proteins are usually XVPT (X represents V/A/L/P) (Zhou et al., 2012a; Zhou and Meier, 2013). Similar to SUN proteins, KASH domain proteins can also form multimers (Djinovic-Carugo et al., 2002; Mislow et al., 2002). The SUN-KASH complexe usually comprise SUN protein trimers and KASH protein trimers. SUN-KASH interactions occur when the KASH domain fits into a hydrophobic pocket that is assembled by three SUN proteins.

### Members and functions of KASH proteins

To date, KASH domain proteins have been identified in various species (Table 2). These KASH proteins are involved in different processes, such as nuclear migration, linkage to the nucleus, attaching nuclei to actin filaments and so on (Table 2).

**Table 2:** Members and functions of the KASH protein family.

**Members**	**Functions**	**References**
**Mammals**		
Syne-1 (Nesprin-1) Syne-2 (Nesprin-2)	Attach nuclei to actin filaments; nuclear migration and nucleokinesis	Apel et al., 2000; Zhang et al., 2007, 2009
Nesprin-3	A versatile connector between the nucleus and the cytoskeleton	Ketema and Sonnenberg, 2011
Nesprin-4	Binding kinesin; cell polarization	Roux et al., 2009
KASH 5	Dynein-driven telomere dynamics in meiosis	Morimoto et al., 2012
***Drosophila***		
Klarsicht	Anchoring microtubules to the NE; nuclear migration and centrosome attachment	Mosleybishop et al., 1999; Patterson et al., 2004; Elhananytamir et al., 2012
MSP-300	Nuclear anchorage	Yu et al., 2006
***C. elegans***		
KDP-1	Cell- cycle progression	Mcgee et al., 2009
ANC-1	Nuclear anchorage	Starr and Han, 2002
UNC-83	Nuclear migration	Starr et al., 2001; Meyerzon et al., 2009
ZYG-12	Links centrosomes to nuclei; meiotic chromosome paring and synapsis	Malone et al., 2003; Sato et al., 2009; Zhou et al., 2009
***S. pombe***		
Kms1	Meiotic dynein-driven chromosome movement and pairing	Miki et al., 2004; Chikashige et al., 2006
Kms2	Meiotic and mitotic chromosome movements	Miki et al., 2004; Chikashige et al., 2006; King et al., 2008
***S. cerevisiae***		
Csm4	Meiotic actin-driven chromosome movements and pairing	Conrad et al., 2008; Koszul et al., 2008
***Dictyostelium***		
Interaptin	Function unknown	Rivero et al., 1998
***Arabidopsis***		
WIP1-3	Anchors WIT1-2 to the NE; anchoring RanGAP to the NE	Yu et al., 2011; Zhou et al., 2012b, 2015b
SINE1	Actin-dependent nuclear positioning	Zhou et al., 2014
SINE2	Contributes to innate immunity against an oomycete pathogen	Zhou et al., 2014
AtTIK	Function unknown	Graumann et al., 2014

The less similarity between KASH domains is very weak, suggesting that many KASH proteins have yet to be discovered. For example, *C. elegans* ZYG-12 and *S. cerevisiae* Csm4 poorly aligns with other KASH domains, but these fit the criteria for KASH proteins (Starr and Fischer, 2005; Conrad et al., 2008; Koszul et al., 2008). Tryptophan–proline–proline (WPP)-interacting proteins (WIP)1-3 and SUN-interacting NE 1-2 proteins (SINE 1-2) are plant-specific KASH proteins that share a low degree of similarity with metazoan KASH proteins (Graumann et al., 2010; Oda and Fukuda, 2011; Zhou et al., 2012a, 2014; Zhou and Meier, 2013). These proteins reside in the ONM via SUN-KASH interactions, fulfilling the criteria for KASH proteins mentioned. AtTIK is a novel *Arabidopsis* KASH domain protein that has been identified using a split-ubiquitin-based membrane yeast two-hybrid screen (Graumann et al., 2014).

Among these reported KASH proteins, only mammalian KASH5, *C. elegans* ZYG-12, and yeast Kms1, Kms2, and Csm4 have been confirmed to be involved in meiosis (Table 2).

### Meiosis-specific adaptations involving the NE

Although the NE has a highly conserved basic structure in eukaryotes, it also undergoes meiosis-specific adjustment to facilitate chromosome dynamics. The nuclear lamina is a protein network that is juxtaposed to the INM nucleoplasmic side. It is mainly composed of lamin proteins. In animals, the nuclear lamina undergoes significant modifications in lamin B1 (B-type lamin) and lamin C2 (A-type lamin isoform) during meiosis, and lamin C2 is exclusively expressed in meiotic cells. This implicates that NE is modified to adapt to the requirements of meiosis (Furukawa et al., 1994; Alsheimer and Benavente, 1996). Current understanding of the functions of the nuclear lamina is limited in plants. It has been postulated that the nuclear matrix component protein (NMCP) family members are likely the best appropriate candidates for plant lamins (Ciska and de la Espina, 2014). Fluorescence resonance energy transfer experiments have shown that the N-termini of AtSUN1 and AtSUN2 co-localize with CRWN1, which is a member of the NMCP family in *Arabidopsis* (Graumann, 2014). However, its physical co-localization does not demonstrate that AtSUN1 and AtSUN2 directly or indirectly interact with CRWN1. Investigations on meiosis-specific adjustments with respect to components and functions of the nuclear lamina in plants are limited.

LINC complexes are important components of the NE that also undergo remarkable adaptations to the requirements of meiosis. SUN proteins and KASH proteins are encoded by various genes that are differentially expressed in various cell types and tissues (Roux et al., 2009; Göb et al., 2010, 2011; Frohnert et al., 2011; Kracklauer et al., 2013; Duong et al., 2014). LINC complexes generally exhibit features that involve specific cellular processes. Meiotic chromosomal movements within the NE are driven by cytoskeletal forces that span the double NE and are transferred to the chromosomes via specific LINC complexes (Kracklauer et al., 2013; Yamamoto, 2014). Unique reconstruction of the NE structure and formation of meiosis-specific LINC complexes are required during telomere attachment, movements, clustering, and reposition (Hiraoka and Dernburg, 2009). The meiosis-specific LINC complexes are modulated with respect to their constituent proteins and interaction partners (Table 3). LINC complexes are species-specific. In mice, meiosis-specific LINC complexes are composed of SUN1 and/or SUN2, and KASH5, which promote chromosome pairing and synapsis (Ding et al., 2007; Schmitt et al., 2007; Morimoto et al., 2012). The SUN protein Sad1 directly interacts with a KASH protein Kms1, assembling a functional meiotic LINC complex in *S*. p*ombe* (Miki et al., 2004). The KASH domain protein ZYG-12 as a SUN1-interacting meiotic LINC component in *C. elegans* (Malone et al., 2003). The *Zea mays* SUN protein, ZmSUN3 is necessary for homologous chromosome synapsis, recombination, and chromosome segregation (Murphy et al., 2010; Murphy and Bass, 2012). However, the real meiotic KASH partner of ZmSUN3 remains elusive. In *Arabidopsis*, AtSUN1 and AtSUN2 are both associated with meiosis (Duroc et al., 2014; Varas et al., 2015). At the same time, the *Arabidopsis* genome encodes four KASH proteins, three WIP proteins (AtWIP1, AtWIP2, and AtWIP3) and one AtTIK protein, which all interact with AtSUN1 (Zhou et al., 2012a; Graumann et al., 2014). However, their definitive meiosis-specific functions remain unclear.

**Table 3:** Constituents of meiotic-specific LINC complexes in various organisms.

	**S. *pombe***	***S. cerevisiae***	***C. elegans***	**Mice**	***Arabidopsis***	**Maize**
SUN domain proteins	Sad1	Mps3	Metafin/SUN-1	SUN1, SUN2	AtSUN1, AtSUN2	ZmSUN1, ZmSUN2, ZmSUN3
KASH domain proteins	Kms1, Kms2	Csm4	ZYG-12	KASH5	AtWIP1-3	U

*U, Unidentified*.

### Kinase-associated meiosis-specific modifications of the NE

The meiosis-specific functions of the ubiquitously expressed SUN proteins indicate that SUN proteins undergo post-translational modifications to mediate their meiotic functions. The Polo-like family of Ser/Thr kinase (PLK) of *C. elegans* co-localizes with PCs during meiosis, bringing about aggregation of SUN-1/ZYG-12 within the NE, thereby mediating dynein-driven chromosomal motions (Harper et al., 2011; Labella et al., 2011; Wynne et al., 2012; Rog and Dernburg, 2015). Phosphorylation modifications of the SUN1 nucleoplasmic domain through checkpoint protein kinase (CHK) family members CHK-2 and PLK-2 influence SUN1 motions within the INM during meiosis in *C. elegans* (Penkner et al., 2009; Sato et al., 2009; Labella et al., 2011). A recent study has shown that CHK-2 is a master regulator of meiosis in *C. elegans*, which first phosphorylates PC-binding zinc finger proteins HIM-8 and ZIMs, which in turn recruits PLK-2 to PCs (Kim et al., 2015).

Cyclin-dependent kinases (CDKs) are another group of highly conserved serine/threonine protein kinases that have been detected in various species from yeast to humans and play key roles in regulating the cell cycle and the cell division. During mammalian meiotic prophase I, CDK2 plays a critical role in meiosis-associated telomeric dynamics and meiosis-specific modifications of the NE components (Ashley et al., 2001; Berthet et al., 2003; Ortega et al., 2003; Viera et al., 2009, 2015). In mice, CDK2 mediates the accurate dynamic distribution of SUN1 protein via phosphorylation of SUN1 protein. SUN1 persists at the NE as a cap from the leptotene to pachytene phases in the absence of CDK2 in mice. CDK2 also affects the assembly of the meiosis-specific nuclear lamina. In the absence of CDK2, the distribution of lamin C2, a meiosis-specific isoform of lamin A and LAP2 (lamin-associated protein) are severely impaired, with a complete lack of LAP2 (Viera et al., 2015). However, the possible pathways to determine altered distribution of lamin C2 in meiosis are unknown.

### Meiosis-specific adaptors between telomeres/PCs and LINC

Telomeres link chromosomes to the NE through the LINCs. From yeast to humans, telomeres (or pairing centers in the worm) are always anchored to the NE by specific adaptors in the meiosis prophase I. The linkers connecting telomeres to LINCs are mainly composed telomere-binding proteins. In *S. pombe*, the linker between LINCs and telomeres is mediated by telomeric proteins Taz-1 and Rap-1, and the meiosis-specific proteins, Bouquet1-4 (Bqt1-4) (Chikashige et al., 2006, 2009). In *C. elegans*, chromosomes are connected to the NE through chromosome-specific pairing centers (PCs), instead of telomeres. Accordingly, LINCs tether chromosomes to the NE through PC-specific proteins, ZIM-1, ZIM-2, ZIM-3, and HIM-8 (Phillips et al., 2005; Phillips and Dernburg, 2006; Penkner et al., 2007; Sato et al., 2009; Baudrimont et al., 2010). During meiosis in *S. cerevisiae*, Ndj1 as a meiosis-specific adaptor connects LINCs to telomeres (Conrad et al., 2007, 2008). In mammals, telomere repeat-binding bouquet formation protein 1/2 (TERB1/2) and membrane-anchored junction protein (MAJIN) form a complex, TERB1/2-MAJIN, which serves as a meiosis-specific link between telomeres and LINCs (Daniel et al., 2014; Shibuya et al., 2014, 2015). In addition, meiotic LINCs of mammals are able to interact with meiosis-specific laminae. It is unknown whether meiosis-specific lamina proteins have an effect on telomere connection with LINCs. Currently, how telomeres are modified to mediate telomeric attachment to the NE during meiosis in plants remains unclear.

### Meiosis-specific adaptors between the cytoskeleton and the LINC

Anchoring linkers bridging LINCs and the cytoskeleton are responsible for transferring cytoskeletal forces to the NE, which then mediates meiotic chromosome movements along the NE during prophase I stages that comprise cytoskeleton or associated motor proteins (Koszul and Kleckner, 2009; Kracklauer et al., 2013). The LINC-complex is bound to the actin cytoskeleton via the atypical KASH protein Csm4 and actin in *S. cerevisiae* (Conrad et al., 2007, 2008). The LINC-complex is connected to microtubules (MTs) in the cytoplasm through Kms1 (KASH protein) and dynein light chain-family protein Dlc1 in *S. pombe* (Miki et al., 2002), KASH5, and dynein in mammals (Morimoto et al., 2012; Rothballer and Kutay, 2013), ZYG-12 KASH protein and dynein motors in *C. elegans* (Sato et al., 2009; Wynne et al., 2012), KASH proteins AtWIP-1, AtWIP-2 and a kinesin1-like protein AtPSS1 in *Arabidopsis* (Duroc et al., 2014; Wang et al., 2014).

### An integrated mechanical system transferring cytoplasm forces into meiotic chromosomes

**Figure 3:**
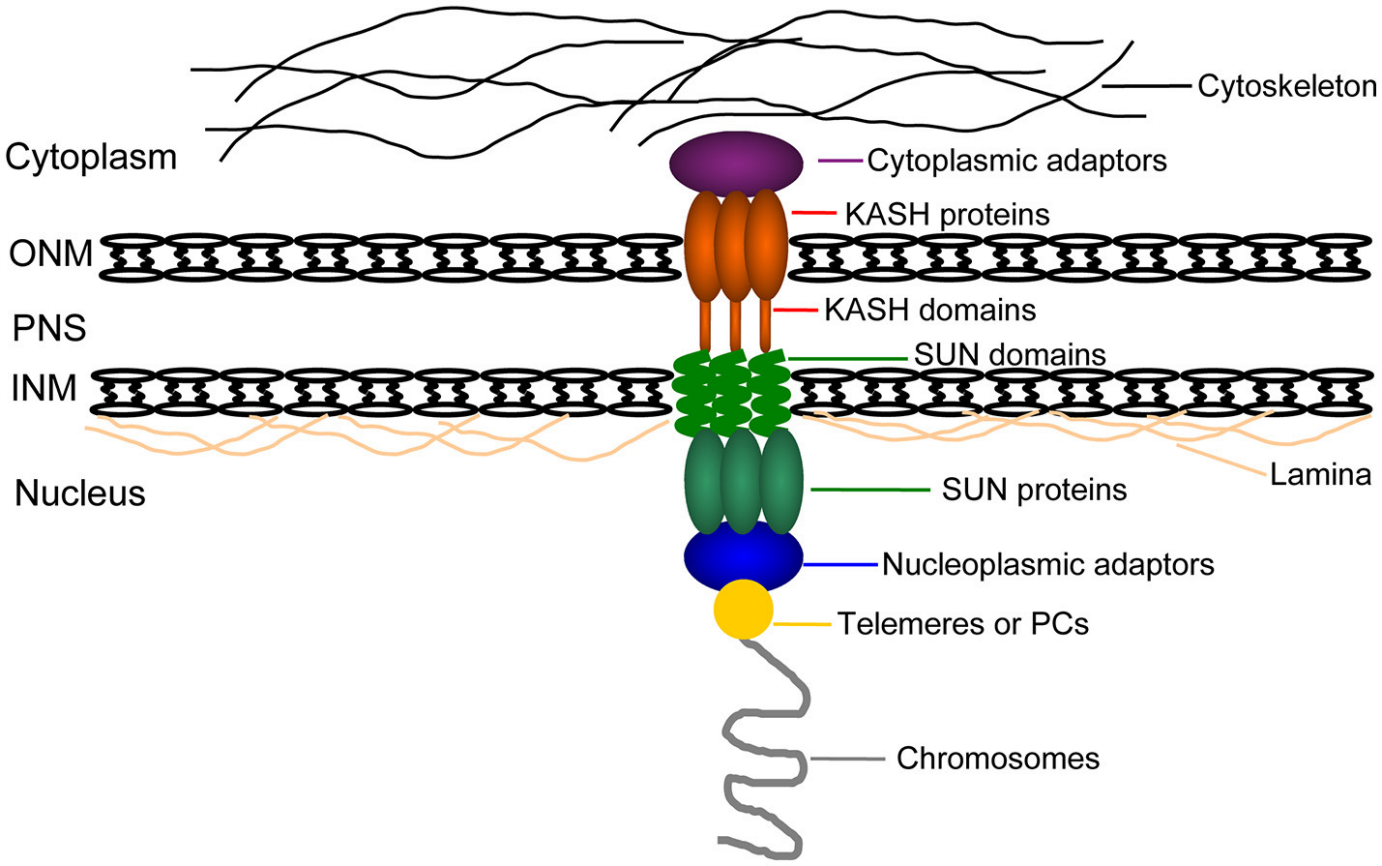
A schematic representation of the link transferring cytoplasm forces into meiotic chromosomes. Telomeres or PCs (gold circle) connect to the NE through nucleoplasmic adaptors (schematized with a blue oval) and the nucleoplasmic domains (in green ovals) of SUN-domain proteins spanning the INM (in green; shown as a trimer). KASH domain proteins span the ONM (in red; shown as a trimer). Then SUN domains (in green helix) can interact with KASH domains (in red stub) in the PNS. Cytoplasmic adaptors (in purple) connect the cytoplasmic domains (in red ovals) of KASH proteins to the cytoskeleton (in black lines). The nucleoplasmic domains of SUN proteins can also interact with lamins (in orange). Cytoskeleton, cytoplasmic adaptors, SUN-KASH protein bridges, nucleoplasmic adaptors and telomeres/PCS form the central link that spans the nuclear envelope, transferring cytoplasm-derived forced into chromosomes. NE, nuclear envelope; INM, inner nuclear membrane; ONM, outer nuclear membrane; PNS, the perinuclear space.

The mechanisms responsible for dynamic chromosome movements have been partially deciphered in model organisms (Figure 3). The LINC complex couples the microtubule network and chromosomes. Nucleoplasmic adaptors tether telomeres or PCs (in *C. elegans*) to LINCs. Cytoplasmic adaptors connect cytoskeleton or cytoskeleton-associated proteins to LINC. The network between the cytoskeleton and chromosomes is telomeres/PCs-nucleoplasmic adaptors-NE-cytoplasmic adaptors-cytoskeleton. The molecular link system by which these forces are implemented differs in constituents in various organisms, telomeres-Taz1/Rap1/Bqt(1-4)-Sad1-Kms1/2-dynein (Dlc1)-MTs in *S*. p*ombe*; PCs-ZIM(1-3)/HIM8-SUN1-ZYG12-Dynein-MTs in *C. elegans*; telomeres-Ndj1-Mps3-Csm4-actin-actin cable in *S. cerevisiae*; telomeres-TERB1/2/MAJIN-SUN1/SUN2-KASH5-dynein-MTs in mice; and telomeres-?-AtSUN1/AtSUN2-AtWIP1/2-kinesin (AtPSS1)-MTs in *Arabidopsis*. Whether and how NMCP family proteins and modification of SUN proteins are involved in the above molecular link system in plants remain unclear.

## Conclusions and future perspectives

Telomere-led chromosomal dynamics within the NE and mediated by LINCs are pivotal for meiosis and thus fertility. The NE as a regulatory platform is finely modified with respect to its constituents in meiosis. Meiosis-specific adaptations of the LINC components, cytoplasmic linkers, and nucleoplasmic linkers contribute to these movements. Our current knowledge of the LINC network can serve as a starting point for future studies in plants. KASH proteins are not well conserved and thus warrant identification of additional novel family members. There are still a number of issues concerning the meiotic adaptions of the NE that need to be addressed. How are ubiquitously expressed NE components regulated during meiosis? Are plant NMCP family proteins involved in telomeric attachment to the NE, similar to the lamina proteins? Are there more adaptor molecules participating in the LINC network?

## Authors contributions

XY and XZ wrote the manuscript. RY, KL, HG, and JL contributed to the preparation of this manuscript. FL, YW, and GW organized and reviewed the manuscript. All authors have read and approved the final manuscript.

### Conflict of interest statement

The authors declare that the research was conducted in the absence of any commercial or financial relationships that could be construed as a potential conflict of interest.
